# Vinyl thianthrenium-mediated cysteine bioconjugation

**DOI:** 10.1038/s42004-024-01111-8

**Published:** 2024-02-27

**Authors:** Huijuan Guo

**Affiliations:** Communications Chemistry, https://www.nature.com/commschem

## Abstract

Covalent cysteine labeling is an important tool in protein modification, however, current methodologies suffer from limited reactivities and require the prior synthesis of individual derivative reagents. Now, a covalent cysteine labeling method that converts the cysteinyl thiol into episulfonium electrophiles in situ has been developed, enabling reactions with various nucleophiles in one step.

Cysteine is often the target of choice for site-selective bioconjugation of proteins under physiological conditions. Cysteinyl thiol can react with electrophiles carrying various functionalities, but often such prefunctionalized electrophiles require separate, individual preparation. Alternatively, cysteinyl thiol can be transformed into an electrophilic linchpin and then coupled with nucleophiles. However, such bioconjugation requires two independent steps and stronger nucleophiles, significantly limiting the potential applications.

Now, Tobias Ritter from Max-Planck-Institut für Kohlenforschung in Germany and colleagues report an in situ cysteine umpolung, using vinyl thianthrenium salts to transform cysteinyl thiol nucleophiles to reactive episulfonium electrophiles (Fig. 1), enabling their conjugation with various bioorthogonal nucleophiles in a single step (10.1038/s41557-023-01388-7)^1^.

**Fig. 1:**
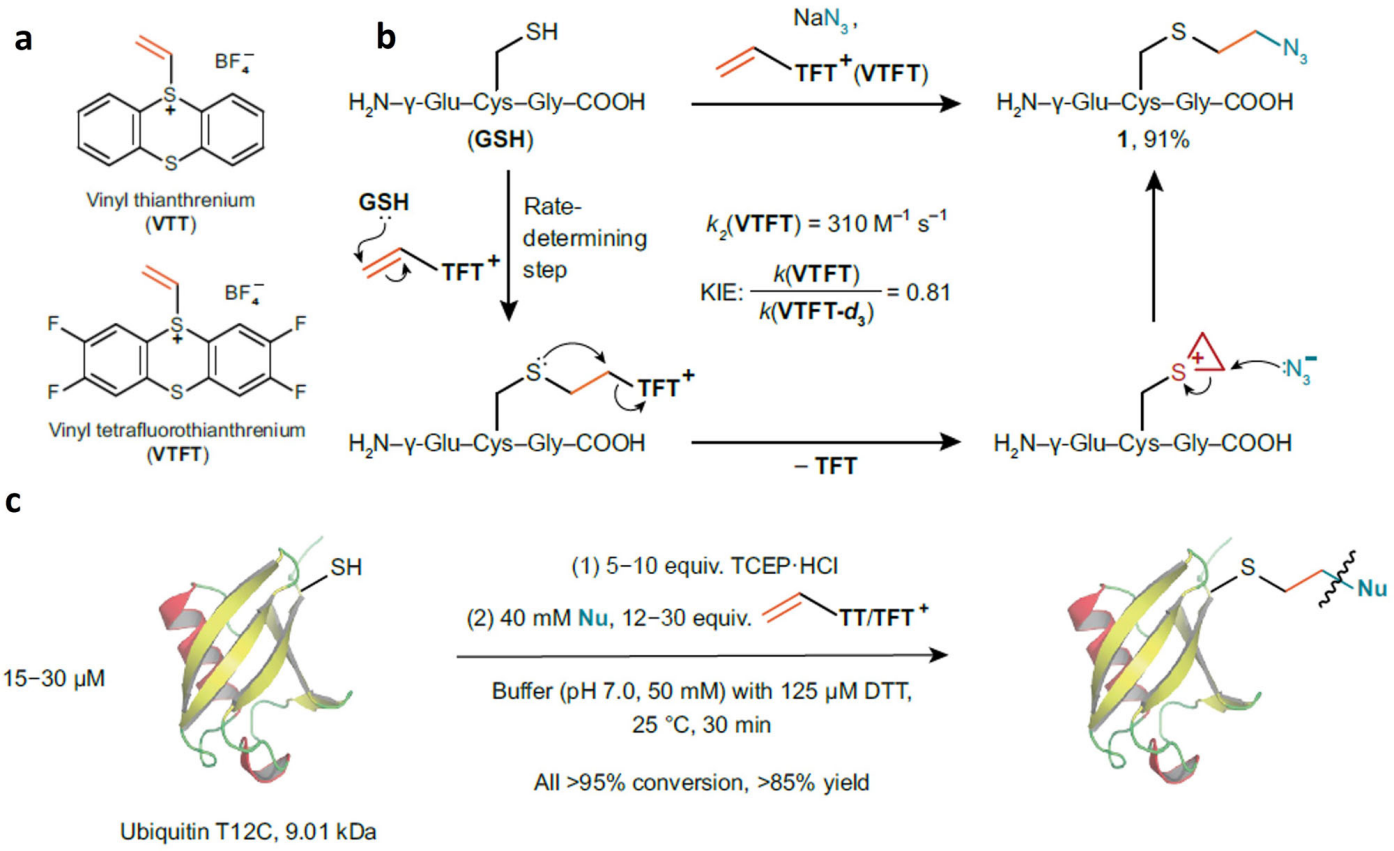
Cysteine labeling by vinyl thianthrenium salts. **a** structures of vinyl thianthrenium and vinyl tetrafluorothianthrenium tetrafluoroborate; **b** formation of episulfonium intermediate; **c** bioconjugation with various nucleophiles. Adapted from ref. ^1^ (10.1038/s41557-023-01388-7).

Thianthrenium chemistry has been applied as a fantastic tool in organic synthesis for site-selective functionalization of various arenes^2^, alkenes, alkanes, alcohols, and amines. Organothianthrenium salts in particular have been developed as attractive precursors for such transformations due to their unique structural properties and reactivites^3^. The team had previously developed a vinyl thianthrenium tetrafluoroborate (VTT) as a distinct vinylating reagent and applied it to various reactions in organic synthesis^4^. They also identified that VTT and vinyl tetrafluorothianthrenium tetrafluoroborate (VTFT) are useful for thiol alkylation, working as reactive Michael acceptors and leaving groups and ensuring displacement of alkylsulfonium species to episulfonium intermediates, which are in turn reactive towards various nucleophiles. The team was motivated to translate this knowledge gained from conventional organic chemistry and apply it to protein chemistry.

Indeed, introducing peptides and proteins to VTT (or VTFT) and sodium azide was shown to result in the formation of azidated peptides and proteins under biocompatible reaction conditions. This azidation exhibits a faster reaction rate than cysteine alkylation with iodoacetamide, a better site selectivity and specificity than for *N*-methylmaleimide, and higher cysteine coverage than for iodoacetamide or tetrafluoroalkyl benziodoxoles. The reaction can additionally be followed using quantitative proteomics by exploiting [^2^H_3_] VTT and [^13^C_2_] VTT isotopologues. The team demonstrated that this labeling reaction can be extended to various bioconjugation nucleophilic reagents, such as isotopic labels, cross-coupling precursors, post-translation modification mimics, infrared labels, and affinity tags. Containing a unique two-carbon linker, VTT and VTFT could also be applied in peptide stapling and peptide macrocyclization through intramolecular reactivity of the episulfonium intermediate with nucleophilic amino acids, as well as in protein–protein cross-linking via intermolecular Cys–Cys linkages.

“Our most significant finding is the distinct reactivity of the new reagent when compared to all other known bioconjugation reactions,” comments Ritter. “Moving forward we are trying to push this reagent to discover previously unappreciated protein–protein interactions,” he concludes.
